# Profiling Children Sexual Abuse in a Sample of University Students: A Study on Characteristic of Victims, Abusers, and Abuse Episodes

**DOI:** 10.3390/ijerph18094610

**Published:** 2021-04-27

**Authors:** Angel Castro, José David Moreno, Berta Maté, Javier Ibáñez-Vidal, Juan Ramón Barrada

**Affiliations:** Facultad de Ciencias Sociales y Humanas, Universidad de Zaragoza, 44003 Teruel, Spain; castroa@unizar.es (A.C.); josedavid.moreno@unizar.es (J.D.M.); matecalvoberta@gmail.com (B.M.); javieriba@cop.es (J.I.-V.)

**Keywords:** children sexual abuse, victims, abusers, episode characteristics, university students

## Abstract

Because of its prevalence and its potential negative consequences, child sexual abuse (CSA) is a public health problem in every country in the world. Knowledge of the characteristics of abuse episodes (victim, abuser, relationship between them, type of sexual contact, duration, threat and/or use of force) is essential to specify the contents of intervention programs for the detection and reduction of the negative consequences of CSA. Starting with an initial sample of 1605 university students of both sexes (70.9% women, 29.1% men), aged between 18 and 26 years (*M* = 21.1, *SD* = 2.2), 90 participants who had suffered an episode of CSA up to age 16 were selected (84.4% women, 15.6% men; *M*_age_ = 21.1, *SD* = 2.2). It was found that: (1) there was a higher prevalence of CSA among women and that the victims’ average age when they suffered abuse was around 11 years; (2) the abusers were mostly male, close to the victims, and with an average of slightly less than 30 years; and (3) there was a significant proportion (25.6%) of CSA cases with penetration and in which force was used or threatened. These results are relevant, as they show that there is still a noteworthy prevalence of CSA cases on university campuses that needs to be addressed. Knowing the characteristics of these episodes is critical to implementing more effective interventions.

## 1. Introduction

Child sexual abuse (CSA) is a global public health problem and can have multiple and varied negative consequences for the victim throughout their life. The Centers for Disease Control and Prevention (CDC) define CSA as any completed or attempted sexual act, sexual contact with, or sexual exploitation of a child [1]. This implies the involvement of a child in a sexual activity that he/she does not fully comprehend, is unable to give informed consent to, or for which the child is not developmentally prepared and cannot give consent, or that violate the laws or social taboos of society [2].

The prevalence of CSA varies depending on the geographical and cultural context in which the studies are carried out, as well as the group being evaluated and the methodology followed, with a high variety of results and difficulty in characterizing the CSA phenomenon [3]. However, there are some global prevalence data. Systematic reviews in recent years have concluded that between 10 and 12% of children will suffer some form of sexual abuse before the age of 18 [4,5,6,7,8,9]. There are some specially studied groups concerning CSA, such as university students, due to the easy access to these samples and the relevance of studying sexual phenomena at this stage, which is characterized by openness and new experiences [10]. Studies among university students have found prevalence rates similar to those of the general population, even higher in some cases [11].

In recent decades, CSA has attracted great interest, both in the scientific community and society at large, due to its prevalence and its short- and long-term consequences [12]. In the existing literature, it has been shown that knowledge of the characteristics of the abuse episodes (i.e., victim, abuser, and situational factors) is essential to be able to design and implement effective intervention programs [4,13].

Concerning the victims, although there is no clear consensus, several studies show that CSA is more prevalent in the years of preadolescence, between ages 8 and 13 [4,8,11,14]. Loinaz and Bigas [15] provide a possible explanation for the greater vulnerability at this stage, especially concerning domestic abuse due to the longer time spent at home with less surveillance.

There is a clear consensus in the existing literature about the higher prevalence of CSA cases among girls than among boys. This result is consistent in all types of samples and collectives [14], with between two and three times the likelihood of suffering an episode of CSA by girls than by boys [7,8,9]. However, several authors warn that the proportion of abused children could be greater than that shown by different statistics, as it is possible that, for fear of stigmatization and different aspects related to masculinity, less abuse is communicated when suffered by a boy [13,16].

As for the abuser, it seems clear that in the vast majority of cases, it is a male, as different studies show, with proportions ranging from 75% to 90% of cases [4,6,13,17]. There is no agreement on the most common age of the abusers. Some studies refer to an age range from 30 to 39 years [15], but others greatly reduce that age. For example, Mohler-Kuo et al. [18] and Pereda and Forns [11] indicate that the most common type of CSA is committed against a victim under the age of 13 by an aggressor with a minimum age difference of five years, with cases in which the aggressor is under the age of 18 being very common.

Abusers, especially in the case of girls, usually belong to the child’s close environment and are people she loves [13,18]. Most studies emphasize this finding. For example, Wallis and Woodworth [19] found that there were fewer cases of CSA perpetrated by strangers (40%) than by acquaintances or family members (60%). What does seem clear, as Loinaz and Bigas [15] claim, is that intra-family abuse is often more severe and has more negative consequences for the victim’s life, because of the relationship between victim and abuser, the betrayal of the abuse, and the habitual increased frequency and duration of those episodes. In fact, although cases of abuse committed by strangers are often punctual, in most cases of intra-family abuse, the victims endure the abuse for more than a year until disclosure [19].

As for the type of sexual contact present in CSA cases, the highest prevalence estimates are for non-physical contact abuse (e.g., inappropriate sexual solicitation, indecent exposure), around 30% of cases. It is followed by the CSA with physical contact without penetration (e.g., kissing, touching) and, finally, CSA with penetration [9,18]. However, differences have been found depending on the age of the victim and the relationship with the abuser [11]. Finally, it seems clear that the threat and/or use of force are present in CSA cases [4,6]. In episodes committed up to the victim’s 13th birthday, abusers often use a type of violence that leaves no serious visible wounds, whereas when the victim is between the age of 13 and 18, especially in the cases of females, the use of violence increases and is more visible [11].

As there is currently greater awareness of and sensitization to CSA, it is considered appropriate to regularly update the prevalence data, as well as to know the characteristics of CSA in different samples and different geographical and cultural contexts. Knowledge of the characteristics of CSA is considered to be of great social relevance and would allow more effective intervention programs to be designed and implemented to avoid its potential negative consequences. Thus, the objective of this study was to determine the characteristics of the episodes of abuse (characteristics of the victim, the abuser, type of relationship between them, type and duration of the sexual contact, and threat and/or use of force) suffered by a sample of university students of both sexes.

## 2. Materials and Methods

### 2.1. Participants and Procedure

The present study is part of a larger project that aimed to evaluate CSA and its relationships with sexual behaviors and sexual health among students from a medium-sized university in Spain. A total of 2496 participants were accessed, but only 1905 of them completed the whole survey. Two inclusion criteria were applied: (1) being aged between 18 and 26 years (46 participants excluded, because they were 17 or over 26 years), based on criteria from previous studies with university samples [10,20,21], and because this is the age range most common in university students; (2) to have answered a set of questions about their sexual relationships up to the age of 16, to be able to identify CSA cases (258 participants excluded, because they did not answer that section of the questionnaire). These criteria led to a sample of 1601 students, 70.9% women (*n* = 1135) and 29.1% men (*n* = 466), aged between 18 and 26 years (*M* = 21.10, *SD* = 2.18). From this initial sample, 90 participants (5.6%) were identified as sexually abused up to 16 years (see criteria below). Thus, the final sample was composed of 76 women (84.4%) and 14 men (15.6%), with a mean age of 21.12 years (*SD* = 2.22). Among them, 81.1% reported being heterosexual, 6.7% gay/lesbian, and 12.2% bisexual. Regarding their relationship status, 58.9% reported having a partner at the time of the study, with an average relationship length of 2.23 years.

Participants were recruited employing the university e-mail distribution list for students. Each student received an e-mail explaining the purpose of the study, the inclusion criteria, contact information of the lead researcher, and the link to the online survey. Only those participants who gave their informed consent could access the survey. This procedure was approved by the Ethics Review Board for Clinical Research of the region (PI13/0114). The data were collected in April 2013.

### 2.2. Measures

#### 2.2.1. Sociodemographic Questionnaire

This questionnaire was used to gather information about gender (women, men), age, sexual orientation (heterosexual, homosexual, or bisexual), and whether they were in a relationship (and if they were, for how long).

#### 2.2.2. Child Sexual Abuse

CSA was assessed in three ways, following the criteria of self-identification as abused, age discrepancy, the use of the force, and the recommendations of different authors [22]. First, participants were asked if they had had sexual relationships up to the age of 16. Those who answered “yes” were asked about their relationships with a maximum of 10 different people with whom they had sexual experience up to that age. For each person, they reported their age, the other person’s age and sex, the type of relationship with these people, the type of sexual contacts, the mean duration time of these contacts, and whether or not threat or force was used. Second, participants were also asked whether or not they had been sexually abused up to the age of 16. Those who answered “yes” were asked about the nature of their experience (their age, other person’s age and sex, the type of relationship with these people, the type of sexual contacts, the mean duration time of these contacts, and whether or not threat or force was used). Finally, participants were considered sexually abused if: (a) they reported having been sexually abused up to the age of 16; (b) they had sexual relationships when they were 12 years old or younger and the other individual was 5 or more years older; (c) the participant was between 13 and 16 years old and the other person was 10 or more years older; or (d) the participant was 16 or younger and the other person threatened or forced him/her.

### 2.3. Data Analyses

As the main objective of the study was to analyze the principal characteristics of the CSA episodes suffered by the participants, descriptive and frequency analyses of the variables were performed. All the analyses performed in this study were run with IBM SPSS Statistics 26.

## 3. Results

Results shown below can also be found on Table 1 and Table 2. First, with respect to the prevalence of CSA found, as already reported in the Materials and Methods section, 5.6% (*n* = 90) of the participants of the initial sample (*n* = 1601) had suffered an episode of CSA. It should be noted here that 4.4% (4/90) of the participants who had suffered CSA reported having suffered it by more than one person, with two abusers in three of the cases and three abusers in the other case. Each of these cases was treated as a new case of abuse, analyzing its characteristics in the same way as in other cases. Different sex prevalence rates were found for the participants, with CSA suffered by 6.7% of the females (76 out of 1135) and by 3.0% of the males (14 out of 466). These differences were statistically significant, indicating that being a woman [*χ*^2^ (1) = 8.49, *p* = 0.004, *r* = 0.07] was related to the experience of CSA.

As for the criteria used to identify CSA cases, 80% (72/90) of the participants who had experienced an episode of abuse identified themselves as victims, 68.9% (62/90) met the age discrepancy criteria, and 45.6% (41/90) met the criterion of having suffered threats and/or the use of force. Of the participants, 41.1% (37/90) met two of these criteria, 32.2% (29/90) met only one of them, and 26.7% (24/90) met all three criteria for being identified as CSA victims.

The victims, as mentioned, were mostly females (84.4%, 76/90 vs. 15.6%, 14/90, males), and the average age at which they suffered the CSA was 11.1 years (*SD* = 3.52), with a range of 4 to 16 years and with a higher proportion of abuse suffered between 7 and 10 years and, above all, between 13–16 years. For their part, the abusers were mostly males, in 94.4% of cases (85/90); they were in the age range 7–70 years; and their average age was 27.8 years (*SD* = 16.94). The age range in which the higher proportion of abusers was found was 11–20 years. The duration of the abusive relationship was in the range of 0 to 99 months, and almost half of the identified CSA cases had a duration of between 1 and 3 months. Additionally, 26.7% (24/90) of the abused participants indicated a duration of 0 months, so it can be interpreted as a single contact. Among the rest, an average duration of the abusive relationship of 9 months (*SD* = 17.41) was found. These results can be found in Table 1.

Concerning their relationship with the victim, 24.4% (20/90) were family members, 22.2% (20/90) were strangers, 21.1% (19/90) were acquaintances but not close to the victim, 18.9% (17/90) were friends of the victim, and 13.3% (12/90) were close friends of the family. Among the intra-familiar abusers, cousins (9/22; 40.9% of these type of abuse), siblings (5/22; 22.7%), and grandparents (4/22; 18.2%) stand out.

The question about the type of sexual contact present in the abusive episode was multiple-response, and the participants could mark as many options as needed. The most common types of contact were sexual touching, marked by 58.9% (53/90) of the abused participants; showing genitalia or asking for genitalia to be shown, indicated by 31.5% (28/90) of the participants; sexual contact with penetration (25.6%; 23/90); and the request for sexual contact, as well as sexual contacts without penetration (e.g., oral sex, masturbation), both indicated by 21.1% (19/90) of the participants. In addition, 20.0% (18/90) indicated that the abuse consisted of unwanted kisses and hugs. Finally, 45.6% (41/90) of the abused participants indicated that they experienced threat and/or use of force, whereas 54.4% (49/90) denied this. These results can be found in Table 2.

## 4. Discussion

CSA continues to be a public health problem present in all countries worldwide and can have devastating consequences for the person suffering it. Studies show that knowing the characteristics surrounding abusive episodes is essential to be able to design and implement more effective intervention programs that prevent or attenuate their negative consequences [4,13]. Therefore, the main objective of this study was to analyze the characteristics of CSA (characteristics of the victim, the abuser, type of relationship between them, type and duration of sexual contact, and threat and/or use of force) suffered by a sample of university students of both sexes.

Having met this objective, some relevant conclusions can be drawn. The first is in terms of the prevalence of CSA found, which was lower than that of other studies carried out both among university students [11,23] and in the general population, recently collected in several systematic reviews and meta-analysis [4,5,6,7,8,9]. There may be various reasons for these differences, although the explanations are partial. The studies share similar methodology to evaluate CSA and were conducted in similar contexts [11,23], but they found prevalence rates that in many cases were double or more the 5.6% obtained in this study.

Perhaps the most convincing explanation is that, in previous studies, it seems that all the participants answered the same number of questions, regardless of whether or not they had suffered CSA. In this study, this did not occur; those who had had sex up to the age of 16 had to provide a lot of information about those relationships, which made their questionnaire much longer. This can lead to increases in missing responses from people who have suffered CSA. It can also be argued that the cohorts of students evaluated between previous studies and ours are different and that the prevalence of CSA may have declined in those years due to increased social awareness. Finally, some authors, such as Stoltenborgh et al. [7], argue that the results indicate that studies with better methodological qualities find lower prevalence rates, which is a god starting point. However, there is no clear explanation for the low prevalence found compared to other studies, and further research should be done in this regard. A CSA rate of 5.6% is still very high and reveals the need to implement a better CSA case detection system, including in universities, and better tools for prevention and intervention in this problem.

As for the characteristics of CSA victims and abusers, results similar to those of the existing literature were found. While the mean age at which abuse was suffered was 11 years, as other studies have noted [4,8,11,14], the higher proportions were found for those over 13 years. A higher prevalence of CSA among females (6.9%) than among males (3.0%) was also found, which also supports the results of previous research [7,8,9]. These findings have two relevant implications. First, that more attention should be paid from all areas (e.g., family, education, health, social) since late childhood and preadolescence. Second, girls remain the main victims of abusive behavior, and other measures must therefore be implemented to try to improve their situation while still paying attention to boys’ vulnerability.

Abusers, for their part, are mostly male (around 95%), a proportion that exceeds even the higher values of the intervals indicated in previous reviews on the subject [4,6,17]. While their average age was around 30 (27.8), nearly half of abusers were between 11 and 20 years. Concerning the age of abusers, there is less agreement in the literature consulted, and the age ranges of the abusers in this study are very broad, between the ages of 7 and 70, so that no clear conclusions can be drawn in this regard. However, with the results obtained, two types of hypothesis can be taken into account. On the one hand, those of authors such as Mohler-Kuo et al. [18] and Pereda and Forns [11] referred to the fact that most cases of CSA are committed by people of relatively close ages to those of victims and, in many cases, under 18 years of age. On another hand, the explanation of Loinaz and Bigas [15] proposed that the average age of abusers was somewhat older (30–39 years). Age variability makes it difficult to design interventions aimed at abusers, but it is clear that they must be implemented and that males must be targeted to prevent abuse, teaching them not to abuse and avoiding blaming the victims and/or potential victims.

In this regard, it has been found that there is a higher proportion of episodes of CSA committed by acquaintances and/or people who are close to the victim than by strangers. Adding the proportion of abusers who are friends of the victim or family (32.2%), family members (24.4%), and acquaintances (21.1%), it is found that three out of four episodes of CSA are committed by people known by the victims. The existing linkage with the abuser seems to be a key issue when intervening. Unfortunately, as it appears in this study and the conclusions of other previous works [13,18,19], there is a high prevalence of episodes of CSA perpetrated by acquaintances and relatives, which often leads to more negative consequences for the victim, because to the abuse itself is added the feeling of mistrust and betrayal, as well as the greater frequency, duration, and severity of the abusive behaviors [15].

As for the type of sexual contact present in CSA episodes, with some specific differences, the results are similar to those of previous studies. It is noted that there is a remarkable prevalence of non-physical-contact abuse (31.5% request or showing genitalia, plus 21.1% requests for sexual contact) and that sex with penetration appears in about 25–30% of the cases of abuse, as other authors have already shown [8,9,18]. Interventions should target all types of abusive behavior, but perhaps more attention should be paid to penetrative relationships, as they can have health and life consequences for abused people, beyond the traumatic experience of the abuse, such as sexually transmitted infections or unwanted pregnancies [14]. The same is true of the use of force or the threat of its use, present in almost half of the cases of abuse identified in this study and which should become a central element of interventions that are universally designed and implemented.

## 5. Conclusions

The study has several limitations that should be considered when interpreting the results. First, it should be emphasized that the data are part of a broader research project that aimed to analyze the prevalence and characteristics of CSA in a sample of a students of a medium-sized Spanish university, as well as its relationships with sexual behaviors and sexual health. For this reason, there are some limitations to this project, especially those relating to the formation of the sample and the generalization of the results. A single university sample was available, eminently feminine and heterosexual, making it difficult to extrapolate the results to the university student collective and more so to non-college youth.

Secondly, we must mention the validity of the data, as they were collected in 2013, so the conclusions must be interpreted cautiously and with an interval of several years. Despite this, these data are up to date compared to other studies carried out in the Spanish population [11,23]. Third, the longer extension of the questionnaire that people who had suffered an episode of CSA had to fill out may have limited the participation of some victims. Concerning the statistical analysis carried out, it should be indicated for information purposes that no inferential statistics were performed, as the objective of the study could be adequately covered by descriptive analysis. Finally, the study shares some limitations with the existing literature on CSA, related to the difficulty of evaluating such an intimate and impactful phenomenon in peoples’ lives, as mentioned by Goldman and Padayachi [3] and discussed in the introduction of this study. Future studies of this topic should try to address these limitations and provide more up-to-date information, which would allow one to analyze the evolution of the prevalence of CSA among university students.

Despite these limitations, the study is considered to make important contributions as it allows us to characterize episodes of CSA suffered by university students, from the points of view of both the victim and the abuser, and the type of relationship and contact established. There are three conclusions: (1) CSA can be suffered at any age, although it has a higher prevalence for preadolescents and adolescents, and abuse is suffered more by girls than by boys; (2) the abusers are usually people close to victims, usually male, of all ages, but in many cases not far from the age of victims; and (3) there are different forms of abuse, but penetration and use of force, or the threat of its use, are very present in them.

This information is essential, it has important present and future implications, and can help different groups. First, it can help other researchers because the existing literature on the characteristics of CSA has been expanded in an understudied population, such as Spanish university students, and following the methodology of other studies carried out in different contexts, which facilitates the comparison of the results. Regarding the current applicability of the results, they are useful for clinicians working with CSA victims/survivors because they will be able to guide their work according to the characteristics of the abuse suffered by their patients, performing differential interventions depending on the characteristics of the victims when they suffered these abuses; the abusers; and the context (i.e., relationship between them, type of contact, duration, and use of force).

Finally, the conclusions provided have several future implications and applications: (1) for health prevention and promotion professionals, who may propose, design, and implement more effective intervention programs both for victims and aggressors; (2) for decision-makers in different institutions related to child protection; (3) for the schools and families of the children, which may have more information about this phenomenon and its characteristics; and (4) for lawyers and other judicial professionals, so they will know how to address this problem in their daily practice. Only with the collaboration of all these groups and, above all, with adequate prevention (so that CSA does not occur) and intervention (once it occurs) can the impact of sexual abuse on children and its impact on the lives of millions of people around the world be reduced.

## Figures and Tables

**Table 1:** Characteristics of sample, victims, abusers, and abuse.

Sex of the Initial Sample	
Women	0.71
Men	0.29
**Sex of the Victims**	
Women	0.84
Men	0.16
**Sex of the Abuser**	
Women	0.06
Men	0.94
**Age when Abused**	
Mean (SD)	11.1 (3.5)
3–4 years	0.03
5–6 years	0.11
7–8 years	0.13
9–10 years	0.18
11–12 years	0.10
13–14 years	0.21
15–16 years	0.23
**Age of the Abuser**	
Mean (SD)	27.8 (17.0)
7–10 years	0.04
11–20 years	0.43
21–30 years	0.17
31–40 years	0.19
41–50 years	0.04
51–60 years	0.07
61–70 years	0.06
**Duration of the Abuse (months)**	
Mean (SD)	9.0 (17.4)
0 months	0.27
1–3 months	0.46
4–6 months	0.11
7–9 months	0.02
10–12 months	0.03
>12 months	0.11

SD = Standard deviation. When not otherwise specified, results correspond to proportions.

**Table 2:** Proportions of relationship among victim and abuser, type of sexual contact, and threat/use of force.

Relationship among Victim and Abuser	
Family members	0.24
Cousins	0.10
Siblings	0.06
Grandparents	0.04
Parents	0.02
Stepparents	0.02
Strangers	0.22
Acquaintances	0.21
Friends of the victim	0.19
Friends of the family	0.13
**Type of Sexual Contact**	
Sexual touching	0.59
Showing/asking genitalia	0.32
Sexual contact with penetration	0.26
Sexual contact without penetration	0.21
Unwanted kisses and hugs	0.20
**Threat/Use of Force**	
Yes	0.46
No	0.54

Note: For the type of sexual contact, as participants could select more than one option, the total proportion was larger than 1.00.
